# Validity and reliability of a wearable-controlled serious game and goniometer for telemonitoring of wrist fracture rehabilitation

**DOI:** 10.1007/s00068-021-01657-5

**Published:** 2021-04-22

**Authors:** Henriëtte A. W. Meijer, Maurits Graafland, Miryam C. Obdeijn, Marlies P. Schijven, J. Carel Goslings

**Affiliations:** 1 Department of Surgery, Amsterdam Movement Sciences, Amsterdam UMC, Location Academic Medical Center, University of Amsterdam, Meibergdreef 9, 1105 AZ Amsterdam, The Netherlands; 2 Department of Surgery, Amsterdam UMC, Location Academic Medical Center, University of Amsterdam, Meibergdreef 9, 1105 AZ Amsterdam, The Netherlands; 3 Department of Plastic, Reconstructive and Hand Surgery, Amsterdam Movement Sciences, Amsterdam UMC, Location Academic Medical Center, University of Amsterdam, Meibergdreef 9, 1105 AZ Amsterdam, The Netherlands; 4 Department of Surgery, Amsterdam Gastroenterology and Metabolism, Amsterdam UMC, University of Amsterdam, Meibergdreef 9, 1105 AZ Amsterdam, The Netherlands; 5 Department of Trauma Surgery, Onze Lieve Vrouwe Gasthuis, Jan Tooropstraat 164, 1061 AE Amsterdam, The Netherlands

**Keywords:** Serious game, Goniometer, Range of motion, Distal radius fracture, Rehabilitation, Telemonitoring

## Abstract

**Purpose:**

To determine the validity of wrist range of motion (ROM) measurements by the wearable-controlled *ReValidate!* wrist-rehabilitation game, which simultaneously acts as a digital goniometer. Furthermore, to establish the reliability of the game by contrasting ROM measurements to those found by medical experts using a universal goniometer.

**Methods:**

As the universal goniometer is considered the reference standard, inter-rater reliability between surgeons was first determined. Internal validity of the game ROM measurements was determined in a test–retest setting with healthy volunteers. The reliability of the game was tested in 34 patients with a restricted range of motion, in whom the ROM was measured by experts as well as digitally. Intraclass-correlation coefficients (ICCs) were determined and outcomes were analyzed using Bland–Altman plots.

**Results:**

Inter-rater reliability between experts using a universal goniometer was poor, with ICCs of 0.002, 0.160 and 0.520. Internal validity testing of the game found ICCs of − 0.693, 0.376 and 0.863, thus ranging from poor to good. Reliability testing of the game compared to medical expert measurements, found that mean differences were small for the flexion–extension arc and the radial deviation-ulnar deviation arc.

**Conclusion:**

The *ReValidate!* game is a reliable home-monitoring device digitally measuring ROM in the wrist. Interestingly, the test–retest reliability of the serious game was found to be considerably higher than the inter-rater reliability of the reference standard, being healthcare professionals using a universal goniometer.

**Trial registration number:**

(internal hospital registration only) MEC-AMC W17_003 #17.015.

## Introduction

Previous research has shown that computer games are a promising adjunct to improve patient motivation in physiotherapy regimens [1–4]. Gamification principles can make rehabilitation exercises more enjoyable and can provide support for patients in a home-based rehabilitation program, hereby supporting treatment adherence [5]. In addition, the implementation and use of wearable sensors for telemonitoring of patients in a home-based rehabilitation setting has long been advocated [6, 7]. The addition of wearable technology to gaming provides an easy way of delivering direct feedback and improving supervision in exercise programs [5, 8].

Distal radius fractures are one of the most frequently occurring types of traumatic injury, making up 18% of fractures in patients presenting to the emergency department, and up to 25% of all fractures in the elderly patient group [9–12]. Distal radius fractures lead to considerable morbidity, resulting in temporary loss of productivity, thus causing a substantial impact on society [13–15]. Physiotherapy or self-supervised exercise programs are commonly recommended as a rehabilitation strategy after such injuries [16–18]. Previous research has shown that practical constraints such as time, costs and travel distance lead to a low adherence to physiotherapy referrals and exercises [19, 20]. Treatment adherence to home-based, self-supervised exercise programs is equally low, mainly due to a lack of monitoring and feedback to patients [21]. Receiving feedback and encouragement is thought to motivate patients, leading to a higher self-efficacy and hereby increasing treatment adherence [22].

To support patients in their rehabilitation process while simultaneously improving patient monitoring, the serious game *ReValidate!* has been developed. The game is controlled by two wearable motion sensors, placed proximally and distally of the wrist, so that they bridge the wrist joint. Each sensor contains an accelerometer, a gyroscope and a magnetometer, enabling three-dimensional registration of movements in the wrist joint. The paired sensors act as controllers for playing the serious game *Revalidate!*, which is installed as a mobile application on a smartphone or tablet. The smartphone or tablet itself acts as the gaming platform. The *ReValidate!* game incorporates telemonitoring of the range of motion (ROM) of the wrist joint and of treatment adherence. In order for the in-game monitoring of patients’ progress to be safe during a clinical rehabilitation program outside of a research setting, and not pose a threat to patient privacy and data safety, the game must comply with medical device regulations [23]. The ROM outcomes as measured by the game application also need to be valid and reliable. Previously, the game has been established to be a promising and valid therapeutic support tool for patients recovering from a distal radius fracture [24]. Other research has established that wearable motion sensors can be used as a reliable method for measuring ROM in patients [25]. The universal goniometer is used as the reference standard, as this is the most frequently and widespread used method of measurement worldwide, next to visual estimation [26]. Newly developed smartphone apps are increasingly researched, and seem to be reliable [27, 28]. The objective of this study was to determine the reliability of active ROM measurements as measured by the wearable-controlled *ReValidate!* mobile game application, compared to the active ROM as measured by an experienced surgeon using a universal goniometer as the reference standard.

## Patients and methods

### Ethics and study design

The study was designed as an observational cohort study. The study was approved by the medical ethical review board of our institution (MEC-AMC W17_003 #17.015).

### Subjects

A total of 34 patients suffering a restricted wrist ROM, and 7 healthy volunteers were recruited for this study. Patient characteristics are shown in the baseline table (Table 1). The patients were asked to participate in the study by their treating physician when visiting the outpatient clinics of the department of plastic, reconstructive and hand surgery, or the department of trauma surgery of our hospital. All patients visited the outpatient clinics for regular follow-ups, varying from 6 week to 1 year after their original injury. Patient all had a restricted range of motion to varying degrees. The seven healthy volunteers with no history of wrist injury or movement restriction due to other disease or factors were recruited from the hospital staff.

**Table 1:** Baseline table with patient characteristics

	Patients (*n* = 34)	Healthy volunteers (* n* = 7)
Age median (IQR)	55 (IQR 34.75–65.25)	30 (IQR 29–30)
Sex female	22 (64.7%)	4 (57.1%)
Conditions	Distal radius fracture: * n* = 12 (35.2%) Conservatively treated: * n* = 7 (20.6%) Operatively treated: * n* = 5 (14.7%) Osteoarthritis: * n* = 13 (38.2%) Ligamentous injuries: * n* = 5 (14.7%) Other chronic wrist injuries: * n* = 4 (11.7%)	Not applicable

*IQR* interquartile range

For the purpose of determining internal validity of the electronic goniometer, sample size calculations were based on the intraclass correlation coefficient. To detect an intraclass correlation coefficient (ICC) of 0.9 when the null-hypothesis is 0.6, using a two-way mixed effects model, with a power of 80% and a significance level of 5%, seven participants were needed. The sample size to test external validity was in agreement with previous studies, and deemed to be sufficient in determining a difference in means of ROM of 5° between ROM measured using the digital game wearable goniometer, the goniometer measurements performed by experienced surgeons, with a significance level of 0.05 and a power of 80% [26, 29, 30]. Though a 5° difference in ROM may not predict clinical relevance, increasing restrictions in range of motion are linked to poorer functional outcomes [31–35]. Variations up to 18° between goniometry measurements performed by different medical professionals have been described previously [34], yet a difference of 5° is also used in comparable studies [27, 34], and was therefore considered to be the maximum for reliable measurements using the *ReValidate!* game application.

### Game setup

The *ReValidate!* serious game is played on a tablet computer (Apple™ iPad®, Apple, Cupertino, California, USA). The game is controlled by the wearable Valedo motion sensors (Valedo®, Hocoma, Switzerland). Alternatively, a version that can be played on a smartphone (Apple™ iPhone®, Apple, Cupertino, California, USA) and that is controlled using the Myo™ Armband (Thalmic Labs, Kitchener, Canada) has been developed to meet the needs of patients not possessing both types of device. As the the Valedo® sensors were previously established to be reliable for range of motion measurements [36], and were more stable in their connection to the mobile application than the smartphone version of the game, only the tablet version of the game was tested for its reliability of range of motion measurements in this study.

**Fig. 1:**
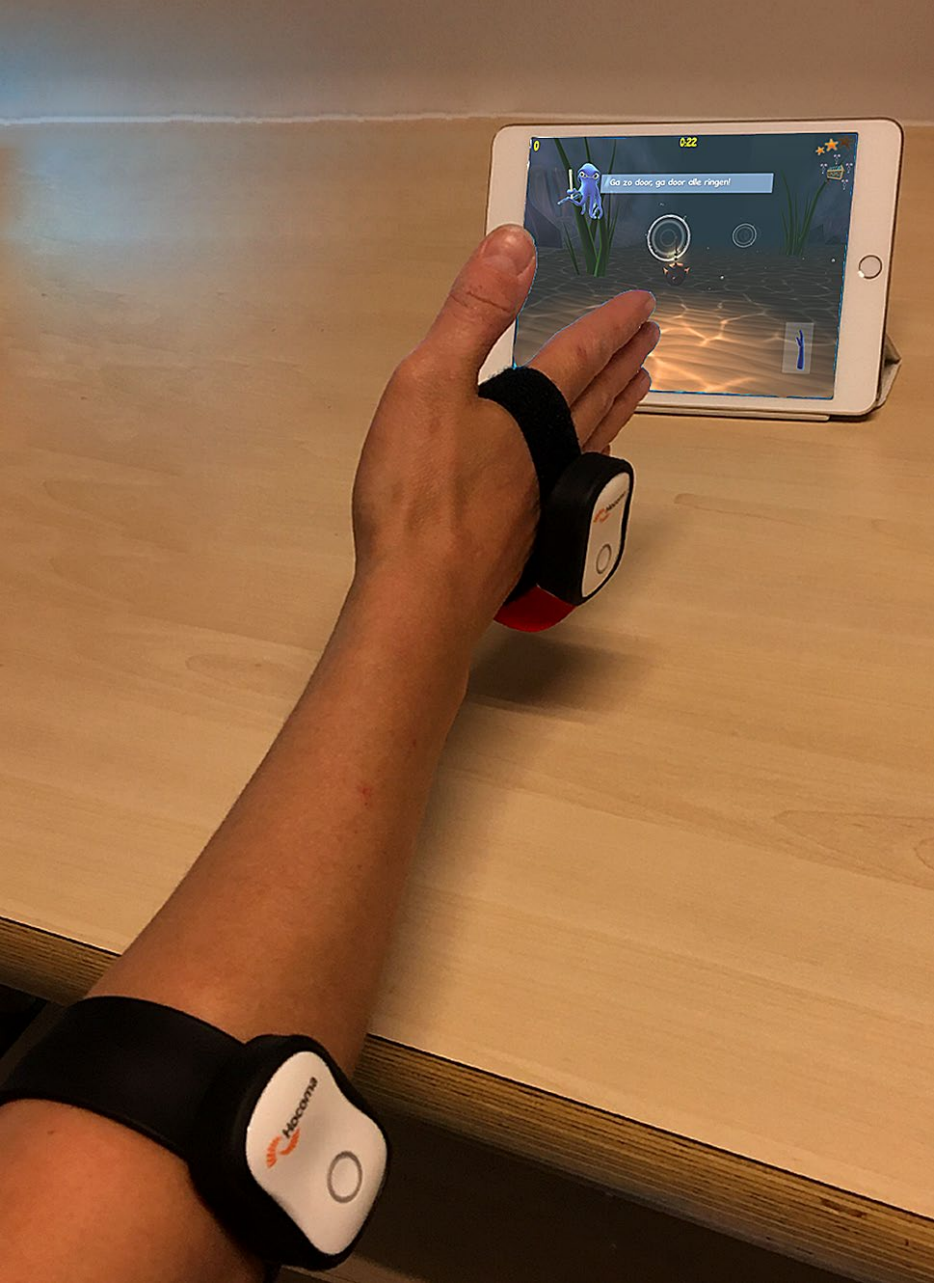
Setup of the Valedo® sensors around dorsum of the hand and lateral side of proximal forearm

Using two separate motion sensors, placed both proximally and distally of the wrist joint, the isolated wrist joint motions can be used as game control, and ROM can be measured. Patients were instructed both orally as well as using pictures within the game, how to attach the sensors to their arm and hand. One sensor is securely strapped to the dorsum of the hand at metacarpal level, while the other sensor is securely strapped to the lateral side of the forearm, just distally to the elbow (Fig. 1), so that the sensors cannot move during gameplay. Sensor placement was checked by the main researcher to ensure correct placement, and corrected if necessary, before continuing to the gameplay session.

**Fig. 2:**
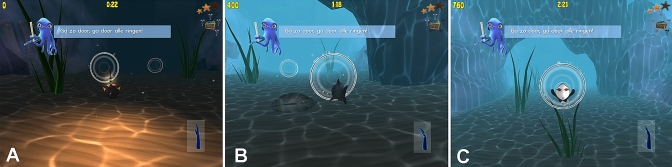
Overview of exercises per movement arc. **a**: pronation/supination (angler fish); **b**: flexion/extension (shark); **c**: radial/ulnar deviation (penguin)

The game is programmed to register both the maximum and average ROM values, as well as the complete motion ‘path’. By moving the wrist, the player controls an underwater avatar (Fig. 2). For each combination of opposing movements (flexion and extension; radial deviation and ulnar deviation; and pronation and supination) a different avatar is controlled. The game consists of 42 levels of increasing difficulty, one for each day of a 6-week rehabilitation program. Each level is completed by steering all three avatars through an underwater parkour successfully. Exercise duration and frequency, average ROM during gameplay, as well as maximum ROM are stored on the device itself, and when connected to the internet, are send to and stored in a secured web-based database. This database is located on the highly secured hospital computer servers, which comply with the General Dara Protection Regulation (GDPR), medical device regulation (MDR) and national and international safety standards including the ISO 27001 and NEN 7510 [37–39]. The data can be retrieved only by patients and healthcare providers using a personal login, and is available for telemonitoring of patients’ progress and recovery.

### Internal (construct) validity measurements of the game

To establish internal validity of the goniometer embedded within the *ReValidate!* game, a test–retest was performed using healthy volunteers. These volunteers were instructed to play the game and to reach their own maximum ROM during gameplay. To ensure the test–retest would not be influenced by confounding factors such as strain or exhaustion resulting from playing the game itself, volunteers were asked to play one complete level of the game twice, with at least 30 min of rest in between. The intraclass correlation coefficient (ICC) between the ROM as measured in the first and second gameplay session was determined using a two-way mixed-effects model (single measures, absolute agreement).

### Inter-rater reliability of experts using a universal goniometer (reference standard) for active ROM measurements

Directly after the two gameplay sessions, four experienced medical professionals were asked to measure the active ROM (flexion–extension, pronation-supination and radial deviation-ulnar deviation arcs) in the healthy volunteers, using a universal (analogue, short-arm) goniometer, as the reference standard. Experts had at least 5 years of experience and used the universal goniometer in their daily practice. To establish if measurements reported by the professionals using a universal goniometer can be considered reliable, the inter-rater reliability between the measurements of the participating professionals was tested. The experts were blinded to previously measured ROM outcomes, both as measured by the game and as measured by the other experts. Participants were instructed not to disclose any previous measurement outcomes. The inter-rater reliability was analyzed using a two-way random-effects model (average measures, absolute agreement).

### External (concurrent) validity

External validity was established by comparing ROM outcomes of the *ReValidate!* game in patients with various levels of restriction in wrist ROM, as measured by highly experienced medical professionals using a universal handheld goniometer. Experts were two plastic surgeons and two trauma surgeons, all of whom were specialized in treating wrist injuries. All experts performed active ROM measurements in their daily practice. First, the maximum active ROM in the affected wrist joint of patients was measured by one of the experts. Subsequently, patients played a single level of the *ReValidate!* game using the same hand and maximum ROM outcomes were extracted from the game database.

Concurrent validity between the game ROM outcomes and the ROM outcomes as measured by the experts was determined using a one-sample t-test to compare mean differences. Values were represented in means and standard deviations, p-values of < 0.05 were considered statistically significant. A difference in means of 5° was considered acceptable for reliable measurements. Scatter-plots and Bland–Altman plots were constructed to evaluate agreement and to assess for any systematic bias.

### Statistical analysis

All analyses were performed using the statistical package for the social sciences (SPSS) version 26 (IBM, New York, USA). ICC outcomes of 0.5 or lower were considered poor, 0.5–0.75 were considered moderate, 0.75–0.9 were considered good, and outcomes larger than 0.9 were considered excellent agreement.

## Results

### Internal validity of the in-game range of motion measurements

The test–retest reliability of the game, reflecting the internal validity of measurement outcomes established by the game was determined by calculating the intraclass correlation coefficient (ICC) using a two-way mixed-effects model (single measures, absolute agreement). Even though patients are instructed to keep their wrist straight when calibrating the game and sensors, the neutral position can vary, since the game lacks visual analysis of movements. Therefore, the motion arcs are represented. Arcs are calculated by adding up the two maximum measurements of motions in opposite directions (flexion + extension; radial deviation + ulnar deviation; pronation + supination). Outcomes are represented in mean ROM and standard deviation, mean difference and ICC and 95% confidence intervals (Table 2).

**Table 2:** Test–retest of mean ROM in degrees as measured within the game

Measurement (°)	Mean (standard deviation)		Mean difference (95% confidence interval	ICC (95% confidence interval)
	Gameplay 1	Gameplay 2		
Palmar-dorsal flexion arc	141.1 (10.7)	142.4 (17.5)	− 1.204 (− 24.829 to 22.420)	− 0.693 (− 1.235 to 0.298)
Pronation-supination arc	118.2 (25.9)	125.9 (19.1)	− 7.671 (− 31.467 to 16.124)	0.376 (− 0.469 to 0.857)
Radial-ulnar deviation arc	94.1 (23.9)	88.6 (16.2)	5.520 (− 3.762 to 14.802)	0.863 (0.444 to 0.974)

Internal validity is represented by the intraclass correlation coefficient (ICC). All data are tested for normal distribution (Shapiro Wilk test = not significant)

Though only the mean difference for the palmar-dorsal flexion is within the predefined 5° range, the mean differences are small, ranging from 1° to 8°. There were no significant differences between the test and retest. Intraclass correlations range from poor (0.376) for the pronation-supination arc, to good (0.693–0.863) for the palmar-dorsal flexion and radial-ulnar deviation arcs.

### Inter-rater reliability between medical professionals

To evaluate the reliability of ROM measurements by experienced professionals using a universal goniometer, the inter-rater reliability was determined. Measurements were registered in degrees. Means and standard deviations between the medical professionals showed large variations, especially in the palmar-dorsal flexion and pronation-supination arcs (ICC 0.16 and 0.02, respectively). Though the measurements showed more agreement in the radial-ulnar deviation arc, there was still a considerable variation and the ICC was only moderate (ICC 0.52). Outcomes of the inter-rater reliability (two-way random-effects model (average measures, absolute agreement)) are represented in Table 3.

**Table 3:** Mean ROM in degrees as measured by the four experts, and inter-rater reliability between the different measurements

Measurements (°)	Mean (standard deviation)				ICC (95% confidence interval)
	Expert 1	Expert 2	Expert 3	Expert 4	
Palmar-dorsal flexion arc	140.5 (18.2)	164.8 (12.4)	156.2 (5.0)	146.4 (4.7)	0.16 (− 0.45 to 0.76)
Pronation-supination arc	188.8 (14.7)	179.2 (4.4)	158.2 (4.6)	162.8 (6.3)	0.02 (− 0.21 to 0.56)
Radial-ulnar deviation arc	79.0 (8.4)	83.5 (18.7)	79.7 (8.7)	72.8 (2.6)	0.52 (− 0.38 to 0.90)

### External validity and reliability

Mean differences were calculated by determining the difference between the ROM measured by the game and measured by the expert (game outcomes–expert outcomes). Mean differences were tested using a one-sample T-test to determine their difference to zero, with zero being the ideal outcome (meaning a perfect agreement between the doctors’ and the game measurements). Outcomes are represented as mean difference, 95% confidence interval of the mean difference and the significance level (Table 4).

**Table 4:** Mean differences between game measurements and measurements by their treating physician using a universal goniometer

Measurements	Mean difference (95% confidence interval)	Significance (*p*)
Palmar-dorsal flexion arc	− 1.5 (− 13.3 to 10.1)	0.788
Pronation-supination arc	66.1 (54.9 to 77.2)	0.000*
Radial-ulnar deviation arc	− 3.6 (− 11.2 to 3.8)	0.325

*Denotes significant difference between values

**Fig. 3:**
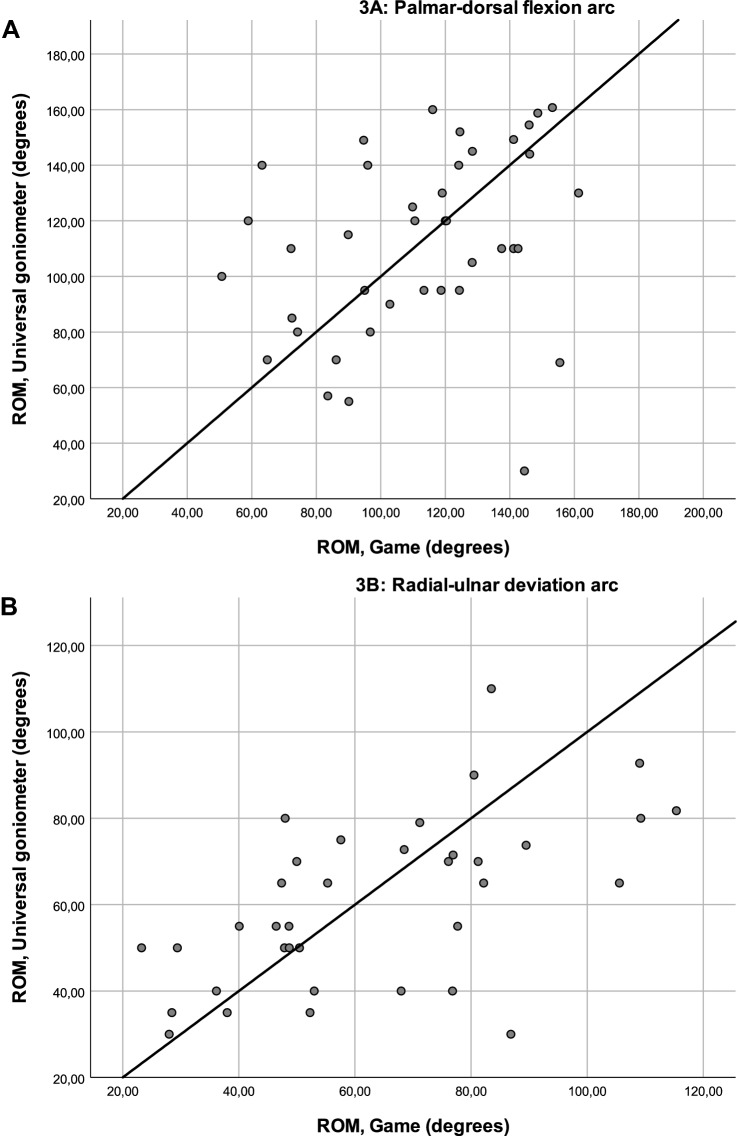
**a, b** Scatter plots of game versus Universal goniometer measurements. **a** Palmar-dorsal flexion arc. **b** Radial-ulnar deviation arc (*ROM* range of motion)

**Fig. 4:**
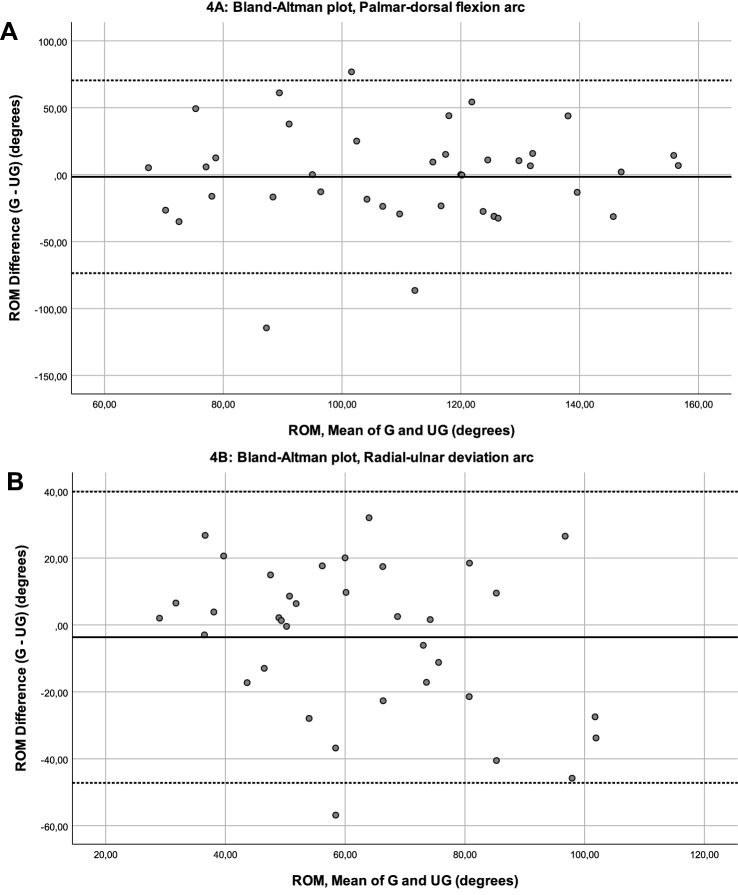
**a, b** Bland–Altman plots of the differences against the means, for the palmar-dorsal flexion arc (**a**), and the radial-ulnar deviation arc (**b**). The bold lines indicate the mean difference, dashed lines denote upper and lower 95% limits of agreement (*ROM* range of motion, *G* game, *UG* universal goniometer)

Scatter plots show the agreement between the two measurement techniques (Fig. 3). Though the scatter plots do not show perfect agreement (line of agreement, bold line), they show comparability between the game and universal goniometer measurements (Fig. 3). Bland–Altman plots provide an insight into measurement distribution and possible systematic bias (Fig. 4). The palmar-dorsal flexion arc plot shows a mean difference of – 1.5 (Fig. 4a; bold line), meaning that the game systematically measures a value 1.5° lower than measured by the experts. The radial-ulnar deviation plot shows a mean difference of –3.6 (Fig. 4b; bold line). The lower and upper 95% limits of agreement are – 71.47 and 72.33 for palmar-dorsal flexion, and – 46.13 and 38.67 for radial-ulnar deviation, respectively (mean ± 1.96 SD; dashed lines in Fig. 4a, b). As these limit of agreement intervals contain zero, this means that there are no differences between the measured values using the game and the universal goniometer. Limits of agreement intervals are wider than the predetermined confidence levels of 5°, however, which can be explained by the large confidence intervals of the game measurements and the expert measurements. The plots show that both methods have a large variation in outcomes, yet there is no systematic bias in either method.

## Discussion

The *ReValidate!* game for wrist rehabilitation has previously shown to be valid in home-based patient care for training active range of motion in patients having suffered a distal radius fracture and has the potential to improve rehabilitation outcomes [24]. To safely use the game as a telemonitoring tool, its ROM measurement outcomes must be reliable when compared to the most widely used method of ROM measurement in clinical practice, being the use of a universal goniometer [27].

This study shows a good test–retest reliability (or construct validity) for the radial-ulnar deviation and palmar-dorsal flexion arcs, as measured during repeated playing sessions of the game. Pronation and supination test–retest outcomes are less reliable and show considerable variation between the gameplay sessions. External validity testing, comparing game outcomes to measurements by experts, shows a good comparability for radial-ulnar deviation and palmar-dorsal flexion arcs. Though the limits of agreement are wider than the ideal level of 5°, the variation can also be attributed to the variation in measurements between medical professionals. The motion sensors (Valedo®, Hocoma, Switzerland) used for controlling the *ReValidate!* game and evaluated in this study, are therefore sufficiently reliable to be used in patient home-based monitoring, compared to ROM measurements as performed by an experienced medical professional.

Regarding monitoring in a home-based rehabilitation setting, the *ReValidate!* mobile game application can be seen as a valid monitoring tool for dorsal-palmar flexion and radial-ulnar deviation, as the mean differences between the game and the medical professionals are within the 4°–6° range. A restriction in ROM in the wrist has been shown in previous studies to be associated with lower functional scores [33, 35]. Remote monitoring of ROM could therefore provide medical professionals with important information on the effectiveness of a rehabilitation program, or warning signs that a patient is at risk for persistent functional limitations [28]. A successful remote monitoring system can hereby help control the increasing demand on healthcare due to the aging population.

It is striking that the participating medical experts, all with at least 5 years of experience and experienced in using a universal goniometer in daily practice, show significant variation in measuring active ROM of the wrist (ICC varying between 0.02 and 0.52). These measurements are the most frequently used type of measurement for wrist range of motion, next to visual estimation, and are therefore considered the ‘gold standard’ by some. As they are used as a reference for the results of the game, these variations inevitably influence outcome of the study. Previous studies show that the inter-rater reliability is dependent on the measurement technique used and the experience and training of the medical professional [29, 40]. Caution is recommended when interpreting measurements made by different professionals, as variation may be larger, and differences of 6°–10° in measurement are described as acceptable [29, 41].

With previous research mainly focused on flexion and extension measurements as a tool for monitoring rehabilitation progress [42], the question remains whether small variations in other ROM measurements should be considered for patient monitoring in a rehabilitation program. The large differences in pronation and supination measurements by the game application, as found in this study, are most likely due to the placement of the motion sensors around the hand and forearm. As the sensor is strapped around the forearm, it will therefore move with the skin as the skin moves relative to the bones. While surgeons use static measurement landmarks (for example, the elbow joint or the upper arm); the game uses a sensor which is not static during pronation and supination movements, hereby leading to smaller measured values for pronation and supination movements. The differences between ROM outcomes as measured by the game application were found to consistently be around 65° smaller than as measured by the surgeons. Therefore, these outcome values could still be used for the purpose of patient follow-up when the game is used as a stand-alone treatment and measurement method in home-based rehabilitation.

While it is a limitation that the game is currently only available for Apple™ (Cupertino, California, USA) devices, this choice was made as Apple™ HealthKit™ (Apple, Cupertino, California, USA) can directly send data to the Epic® electronic health records (Epic Systems Corporation, Verona, Wisconsin, USA). This will allow physicians immediate access to game data, including progress reports and treatment adherence details. Though the smartphone version of the game has not been tested for reliability of goniometric measurements, this version still allows for home-based monitoring of treatment adherence. The game has been developed specifically for compatible smartphones and tablets, and follows the ‘bring your own device’ principle. This principle means that when the game is compatible with more different types of mobile devices, more patients are enabled to practice their rehabilitation exercises using the game. The game will therefore be expanded to be compatible with other types of devices in future development.

It can also be considered a limitation of this study the ROM measurements by experts contain too much ‘human error’. Though these measurements are commonly used to monitor patients’ rehabilitation progress in daily medical practice, they seem too inconsistent be considered to be the gold standard for ROM measurements.

## Conclusions

In this study, experienced healthcare professionals’ measurements show a poor inter-rater reliability using a universal goniometer in measuring active ROM of the wrist. In contrast, the wearable-controlled mobile game *ReValidate!*, which incorporates a digital goniometer, shows a higher reliability and validity in measuring active ROM of the wrist joint. This study shows that medical professionals, including surgeons and hand therapists, can rely on a commercially available off-the-shelf tool to reliably monitor the progress of their patients participating in a home-based rehabilitation program, without requiring hospital visits for monitoring. Especially during a worldwide pandemic, with restricted access to healthcare and where social distancing rules apply, this is an interesting development.

## References

[1] Brichetto G (2013). The effect of Nintendo(R) Wii(R) on balance in people with multiple sclerosis: a pilot randomized control study. Mult Scler.

[2] Joo S, Shin D, Song C (2015). The effects of game-based breathing exercise on pulmonary function in stroke patients: a preliminary study. Med Sci Monit.

[3] Morone G (2014). The efficacy of balance training with video game-based therapy in subacute stroke patients: a randomized controlled trial. Biomed Res Int.

[4] Rand D (2014). Eliciting upper extremity purposeful movements using video games: a comparison with traditional therapy for stroke rehabilitation. Neurorehabil Neural Repair.

[5] Meijer HA (2018). Systematic review on the effects of serious games and wearable technology used in rehabilitation of patients with traumatic bone and soft tissue injuries. Arch Phys Med Rehabil.

[6] Appelboom G (2014). Smart wearable body sensors for patient self-assessment and monitoring. Arch Public Health.

[7] Patel S (2012). A review of wearable sensors and systems with application in rehabilitation. J Neuroeng Rehabil.

[8] Sethi A (2020). Advances in motion and electromyography based wearable technology for upper extremity function rehabilitation: a review. J Hand Ther.

[9] Rozental TD (2002). Survival among elderly patients after fractures of the distal radius. J Hand Surg Am.

[10] O'Neill TW (2001). Incidence of distal forearm fracture in British men and women. Osteoporos Int.

[11] Court-Brown CM, Caesar B (2006). Epidemiology of adult fractures: a review. Injury.

[12] Nellans KW, Kowalski E, Chung KC (2012). The epidemiology of distal radius fractures. Hand Clin.

[13] Angermann P, Lohmann M. Injuries to the hand and wrist. A study of 50,272 injuries. J Hand Surg Br. 1993;18(5):642–4. 10.1016/0266-7681(93)90024-a 8294834

[14] de Putter CE (2012). Economic impact of hand and wrist injuries: health-care costs and productivity costs in a population-based study. J Bone Joint Surg Am.

[15] Larsen CF (2004). The epidemiology of hand injuries in the Netherlands and Denmark. Eur J Epidemiol.

[16] Nederlandse Vereniging voor Heelkunde, *Richtlijn Distale Radiusfracturen: diagnostiek en behandeling.* 2010.

[17] American Academy of Orthopaedic Surgeons, *The treatment of distal radius fractures - Guideline and evidence report*. In: Recommendation 21–23. Rosemont, IL: AAOS; 2009. p. 84–92.

[18] Deutschen Gesellschaft für Unfallchirurgie e.V. (DGU), *Distale Radiusfraktur - Leitlinie Unfallchirurgie*, in *Physiotherapie*. 2014, DGU: Göttingen. pp. 32–33.

[19] Bjork M, et al. Self-efficacy corresponds to wrist function after combined plating of distal radius fractures. J Hand Ther, 2020. 10.1016/j.jht.2020.01.001 32088082

[20] Sluijs EM, Kok GJ, van der Zee J. Correlates of exercise compliance in physical therapy. Phys Ther. 1993;73(11):771–82 **(discussion 783–6)**. 10.1093/ptj/73.11.771 8234458

[21] Jack K (2010). Barriers to treatment adherence in physiotherapy outpatient clinics: a systematic review. Man Ther.

[22] Picha KJ, Howell DM (2018). A model to increase rehabilitation adherence to home exercise programmes in patients with varying levels of self-efficacy. Musculoskelet Care.

[23] European Parliament and the Council of the European Union. Regulation (EU) 2017/745 of the European Parliament and of the Council of 5 April 2017 on medical devices, amending Directive 2001/83/EC, Regulation (EC) No 178/2002 and Regulation (EC) No 1223/2009 and repealing Council Directives 90/385/EEC and 93/42/EEC (Text with EEA relevance). Official Journal of the European Union 2020 24 April 2020 [cited 2020 1 Sept]; Available from: https://eur-lex.europa.eu/eli/reg/2017/745/oj.

[24] Meijer HAW (2019). Face validity and content validity of a game for distal radius fracture rehabilitation. J Wrist Surg.

[25] Nussbaumer S (2010). Validity and test-retest reliability of manual goniometers for measuring passive hip range of motion in femoroacetabular impingement patients. BMC Musculoskelet Disord.

[26] McVeigh KH (2016). Accuracy and validity of goniometer and visual assessments of angular joint positions of the hand and wrist. J Hand Surg Am.

[27] Keogh JWL (2019). Reliability and validity of clinically accessible smartphone applications to measure joint range of motion: a systematic review. PLoS ONE.

[28] Pourahmadi MR (2017). Reliability and concurrent validity of a new iPhone((R)) goniometric application for measuring active wrist range of motion: a cross-sectional study in asymptomatic subjects. J Anat.

[29] Carter TI (2009). Accuracy and reliability of three different techniques for manual goniometry for wrist motion: a cadaveric study. J Hand Surg Am.

[30] Bashardoust Tajali S (2016). Reliability and validity of electro-goniometric range of motion measurements in patients with hand and wrist limitations. Open Orthop J.

[31] Adams BD (2003). Impact of impaired wrist motion on hand and upper-extremity performance. J Hand Surg.

[32] Franko OI, Zurakowski D, Day CS (2008). Functional disability of the wrist: direct correlation with decreased wrist motion. J Hand Surg Am.

[33] Yang Z (2018). Association of wrist and forearm range of motion measures with self-reported functional scores amongst patients with distal radius fractures: a longitudinal study. BMC Musculoskelet Disord.

[34] Reissner L (2019). Minimal detectable difference of the finger and wrist range of motion: comparison of goniometry and 3D motion analysis. J Orthop Surg Res.

[35] Egol KA, et al. Hand stiffness following distal radius fractures: who gets it and is it a functional problem? Bull Hosp Jt Dis (2013) 2014;72(4):288–93. 25986354

[36] Roetenberg D (2019). Comparison of a low-cost miniature inertial sensor module and a fiber-optic gyroscope for clinical balance and gait assessments. J Healthc Eng.

[37] Jansen, M., et al. [Apps in healthcare, what do I need to know?]. Ned Tijdschr Geneeskd, 2020;164. 33201623

[38] European Parliament and the Council of the European Union, *Regulation (EU) 2017/745 on medical devices*, in *2017/745*, European Parliament and the Council of the European Union. 2017, Official Journal of the European Union: Brussels, Belgium.

[39] European Parliament and the Council of the European Union, Regulation (EU) 2016/679 on the protection of natural persons with regard to the processing of personal data and on the free movement of such data, in 2016/679, European Parliament and the Council of the European Union. 2016, Official Journal of the European Union: Brussels, Belgium.

[40] Horger MM (1990). The reliability of goniometric measurements of active and passive wrist motions. Am J Occup Ther.

[41] Scott KL, Skotak CM, Renfree KJ (2019). Remote assessment of wrist range of motion: inter- and intra-observer agreement of provider estimation and direct measurement with photographs and tracings. J Hand Surg Am.

[42] Handoll HHG, Elliott J. Rehabilitation for distal radial fractures in adults (Cochrane review) [with consumer summary]. Cochrane Database Syst Rev 2005;Issue 9, 2015. 10.1002/14651858.CD003324.pub3 PMC9250132 26403335

